# Comparative Efficacy of Adagrasib and Sotorasib in KRAS G12C-Mutant NSCLC: Insights from Pivotal Trials

**DOI:** 10.3390/cancers16213676

**Published:** 2024-10-30

**Authors:** Tzu-Rong Peng, Ta-Wei Wu, Tai-Yung Yi, An-Jan Wu

**Affiliations:** Department of Pharmacy, Taipei Tzu Chi Hospital, Buddhist Tzu Chi Medical Foundation, New Taipei City 23142, Taiwan; tzu.rong@tzuchi.com.tw (T.-R.P.); tawei@tzuchi.com.tw (T.-W.W.)

**Keywords:** NSCLC, KRAS G12C mutation, adagrasib, sotorasib

## Abstract

**Background**: The KRAS G12C mutation, prevalent in various malignancies, including non-small cell lung cancer (NSCLC), represents a unique therapeutic target. Adagrasib and sotorasib, two FDA-approved agents specifically targeting this mutation, have shown promise in clinical trials. This study aims to compare their efficacy in treating KRAS G12C-mutated NSCLC, drawing insights from pivotal clinical trials. **Methods**: We analyzed data from three key clinical trials: KRYSTAL-1, CodeBreak100, and CodeBreak200. Our methodology involved reconstructing individual patient data from published Kaplan–Meier curves using the IPDfromKM tool (Version 0.1.10). The primary endpoints were progression-free survival (PFS) and overall survival (OS), evaluated through hazard ratios (HRs) and the restricted mean survival time (RMST) method. **Results**: The HR for PFS favored adagrasib (HR: 0.90 [95% CI: 0.69, 1.19], *p* = 0.473), suggesting a non-significant trend toward better disease control compared to sotorasib. For OS, the HR was 0.99 [95% CI: 0.75, 1.33] (*p* = 0.969), indicating no significant difference between the two drugs. RMST analysis supported these findings, with adagrasib showing a consistently higher RMST in PFS at 6, 12, and 18 months. However, OS benefits converged over time, with adagrasib marginally surpassing sotorasib by the 18-month mark. **Conclusions**: This comprehensive analysis reveals that while adagrasib may offer a slight advantage in PFS, both drugs demonstrate comparable efficacy in OS for KRAS G12C-mutated NSCLC. The subtle differences observed, particularly in PFS, could inform clinical decision-making, emphasizing the need for personalized treatment strategies. Future research should focus on long-term effects and identifying patient subgroups that may benefit more from one drug over the other.

## Simple Summary

Non-small cell lung cancer (NSCLC) is a leading cause of cancer-related deaths, particularly among patients with a specific mutation in the KRAS gene known as KRAS G12C. Two targeted therapies, adagrasib and sotorasib, have been developed for this mutation. This manuscript reviews three key clinical trials that evaluated the efficacy of these drugs in patients with KRAS G12C-mutated NSCLC. We compare their effectiveness in slowing disease progression and examine their differing side effect profiles, both of which are crucial for treatment decisions. While both drugs demonstrate efficacy, their side effects can influence patient tolerance and overall experience. Understanding these differences enables physicians to make more informed choices about the most appropriate treatment for each patient. Our analysis indicates that, although the overall survival rates for both drugs are comparable, certain patients may derive greater benefits from one therapy over the other, depending on individual characteristics and the management of side effects.

## 1. Introduction

The Rat Sarcoma virus (RAS) signaling pathway is a central regulator of various cellular processes, encompassing integral cascades such as the mitogen-activated protein kinase (MAPK) and phosphoinositide 3-kinase (PI3K) pathways. These cascades modulate cellular proliferation, survival, and differentiation. Dysregulation within these pathways is a hallmark of oncogenesis and cancer progression. Furthermore, the RAS pathway exhibits intricate interactions with other signaling networks, including the receptor tyrosine kinase (RTK) pathway, thereby extending its influence on cellular dynamics [1,2].

Historically, directly targeting RAS proteins has posed a formidable challenge in oncology. The inherent complexity of RAS, combined with the absence of suitable binding sites for therapeutic agents, has rendered it “undruggable” [3]. However, contemporary oncological research has shifted towards devising innovative strategies to attenuate RAS signaling. This includes the development of mutant-selective inhibitors, particularly those targeting the KRAS G12C mutation, as well as the exploration of immunotherapies aimed at RAS-mutant neoplasms [3,4]. The KRAS G12C mutation is notable for its ability to induce a hyperexcitable state rather than a constitutively active one, distinguishing it from other RAS mutations [5]. This unique hyperexcitability makes KRAS G12C an attractive therapeutic target. Research suggests that antagonizing the inactive, nucleotide-bound guanosine diphosphate (GDP)-bound conformation of KRAS G12C presents a promising avenue for anti-RAS drug development [5,6].

Moreover, inhibitors specific to KRAS G12C have shown promise in curtailing the activity of the mutant protein. However, a significant challenge arises from the adaptive feedback reactivation of wild-type RAS, identified as a primary resistance mechanism against KRAS G12C inhibitors [7]. This reactivation is facilitated by signaling from various receptor tyrosine kinases (RTKs) to RAS, mediated through the SHP2 protein. The concurrent inhibition of SHP2 and KRAS G12C has shown potential in counteracting this feedback and enhancing the therapeutic efficacy of KRAS G12C inhibitors [7]. The KRAS G12C mutation, a specific alteration in the KRAS gene, has been identified in a variety of malignancies, including non-small cell lung cancer (NSCLC) and colorectal cancer (CRC). This mutation leads to the constitutive activation of the KRAS protein, promoting dysregulated signaling pathways that contribute to tumor growth and progression [2,8]. Given its prevalence and potential as a therapeutic target, particularly in NSCLC, mutant-specific KRAS G12C inhibitors such as MRTX849 (adagrasib) and AMG 510 (sotorasib) have emerged as promising therapeutic agents, demonstrating efficacy in treating KRAS G12C-driven malignancies [9].

In KRAS G12C-mutant NSCLC, the relationship between PD-L1 expression and targeted therapies is complex. PD-L1 expression can influence tumor immunity, potentially affecting therapy responses. Low PD-L1 levels in KRAS-mutant lung cancer patients may still allow for benefits from immune checkpoint inhibitors, indicating that PD-L1 is not the only factor determining treatment success [10]. The tumor microenvironment (TME) in NSCLC, featuring regulatory T cells and myeloid-derived suppressor cells, can create an immunosuppressive landscape that enhances PD-L1 expression, complicating responses to both KRAS G12C inhibitors and PD-L1 therapies [11,12]. Factors like EGFR mutations and inflammatory cytokines also influence PD-L1 expression and immune responses [13,14].

In light of these developments, understanding the comparative efficacy of sotorasib and adagrasib can help guide treatment decisions and optimize patient outcomes. This knowledge informs clinicians about the potential benefits and limitations of each drug, enabling them to make informed choices based on individual patient characteristics and treatment goals. This study aims to critically evaluate the therapeutic efficacy of the two FDA-approved agents for KRAS G12C-mutated NSCLC, namely adagrasib and sotorasib, by analyzing the results from their pivotal trials.

## 2. Methods

### 2.1. Study Selection and Data Extraction

In this comprehensive analysis, we focused on phase II and beyond (including phase III) clinical trials where adagrasib or sotorasib was administered in at least one arm for NSCLC. A key inclusion criterion was the availability of Kaplan–Meier survival curves as part of the reported outcomes. These trials were specifically chosen for their contribution to the Food and Drug Administration (FDA) or European Medicines Agency (EMA) approval of the drugs in question. In cases where multiple publications stemmed from a single clinical trial, we meticulously selected those reports that provided either extended follow-up durations or encompassed a larger patient cohort. This approach was adopted to address potential issues of data non-independence that could arise from overlapping patient populations.

To reconstruct patient-level data, we employed the IPDfromKM tool, a technique that has gained recognition for its efficacy in deriving individual patient data from published Kaplan–Meier curves [15]. This reconstruction allowed for a more granular analysis of the trial data. We also conducted a thorough comparison of inclusion and exclusion criteria across the selected trials. In instances where individual trials shared similar criteria and employed identical regimens in one of their treatment arms, we pooled the reconstructed patient-level data. This pooling was aimed at enhancing the overall sample size for each drug, thereby bolstering the robustness of our analysis. This methodology aligns with established practices in clinical trial analysis, as noted in previous studies [16].

### 2.2. Endpoints and Statistical Analysis

The primary endpoints for evaluating the efficacy of adagrasib and sotorasib in this study are progression-free survival (PFS) and overall survival (OS). Recognizing that survival curves from different treatment groups, particularly those involving distinct drugs, may not always adhere to the proportional hazards assumption, we opted for a multifaceted analytical approach.

In addition to conventional hazard ratios (HRs) derived from Cox regression analysis, we incorporated the restricted mean survival time (RMST) method into our evaluation [17]. The RMST method offers a valuable alternative perspective, especially in scenarios where the proportional hazards assumption is not met. This dual approach ensures a comprehensive and nuanced assessment of the treatment efficacy, catering to the complexities inherent in oncology clinical trials.

## 3. Results

### 3.1. Overview of Selected Clinical Trials

This study synthesizes findings from three pivotal clinical trials, identified as Janne 2022 [18], Dy 2023 [19], and DeLangen 2023 [20], which investigated the efficacy of adagrasib and sotorasib in treating NSCLC. These trials, namely KRYSTAL-1, CodeBreak100, and CodeBreak200, respectively, offer critical insights into the therapeutic potential of these drugs. Notably, the Kaplan–Meier curves for CodeBreak100 were sourced from Dy 2023, as opposed to Skoulidis 2021 [21], due to the extended follow-up period in the former. A comprehensive comparison of trial designs, treatment regimens, and key inclusion and exclusion criteria is delineated in Table 1. Patient demographics and baseline characteristics are systematically cataloged in Table 2.

### 3.2. Inclusion and Exclusion Criteria Across Trials

A commonality among the KRYSTAL-1, CodeBreak100, and CodeBreak200 trials is the requirement for participants to possess the KRAS G12C mutation and measurable disease as per RECIST guidelines, version 1.1. All three trials uniformly excluded patients who had previously received treatment with a direct KRAS G12C inhibitor. Additionally, participants who had undergone systemic anticancer therapy within specific timeframes before the trial commencement were excluded (2 weeks for KRYSTAL-1 and CodeBreak100, 28 days for CodeBreak200). Similarly, those who received therapeutic or palliative radiation therapy within 2 weeks before the trial initiation were also excluded.

### 3.3. Comparative Analysis and Survival Outcomes

**Figure 1:**
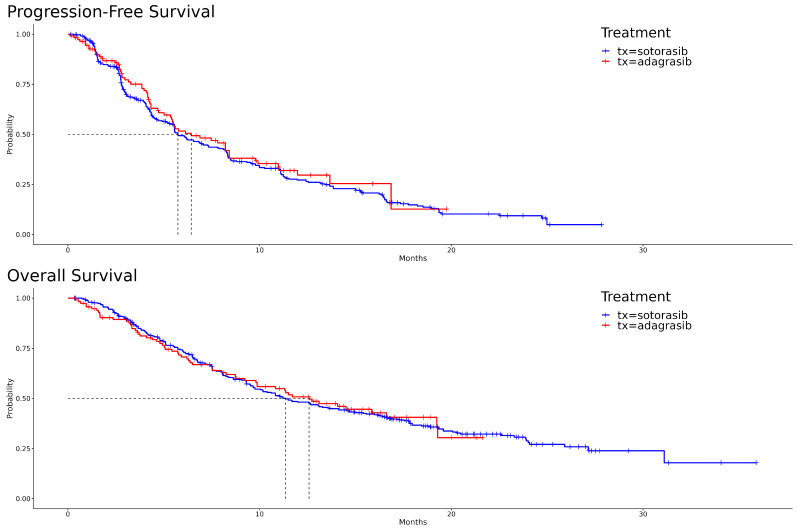
Kaplan–Meier survival curves for adagrasib and sotorasib: progression-free survival (PFS) in the upper panel and overall survival (OS) in the lower panel.

The Kaplan–Meier curves, illustrating PFS and OS for both adagrasib and sotorasib, are depicted in Figure 1. A visual examination reveals that, for PFS, adagrasib’s curve predominantly extends to the right of sotorasib’s. In the initial phase of OS, sotorasib shows marginally superior performance compared to adagrasib. However, this trend reverses between the seventh and eighth months, with adagrasib demonstrating slightly better outcomes. The hazard ratio for adagrasib relative to sotorasib in PFS is 0.90 [95% CI: 0.69, 1.19] (*p* = 0.473), and in OS, it is 0.99 [95% CI: 0.75, 1.33] (*p* = 0.969). The median survival times for PFS are 6.43 months for adagrasib and 5.73 months for sotorasib, while for OS, they are 12.6 months and 11.4 months, respectively. These statistical outcomes suggest no significant differences between the two drugs.

### 3.4. Restricted Mean Survival Time Analysis

**Figure 2:**
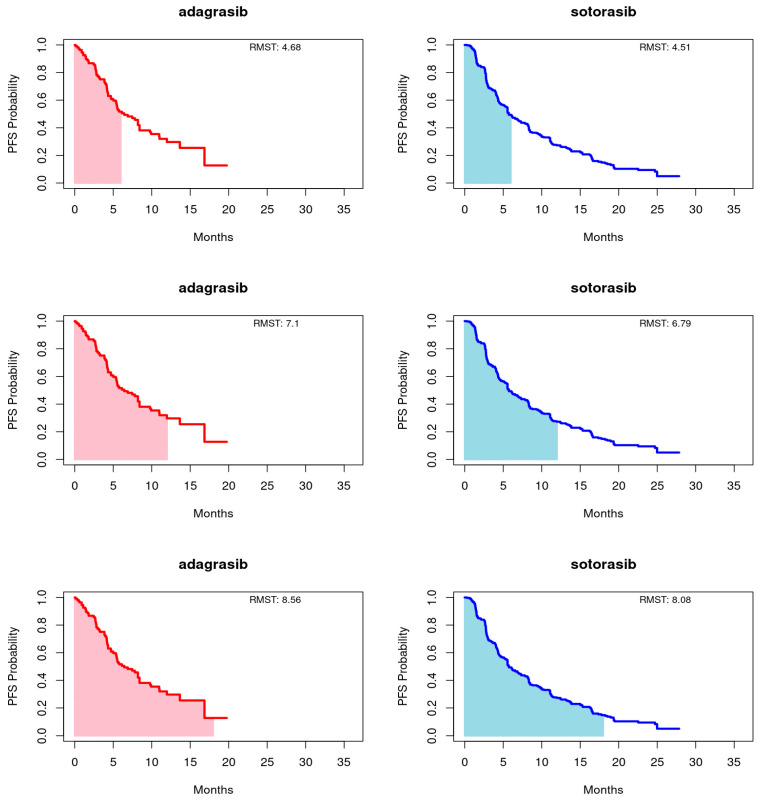
Restricted mean survival time (RMST) of progression-free survival (PFS) for adagrasib (**left panel**) and sotorasib (**right panel**) was evaluated at 6 (**upper panel**), 12 (**middle panel**), and 18 (**bottom panel**) months.

**Figure 3:**
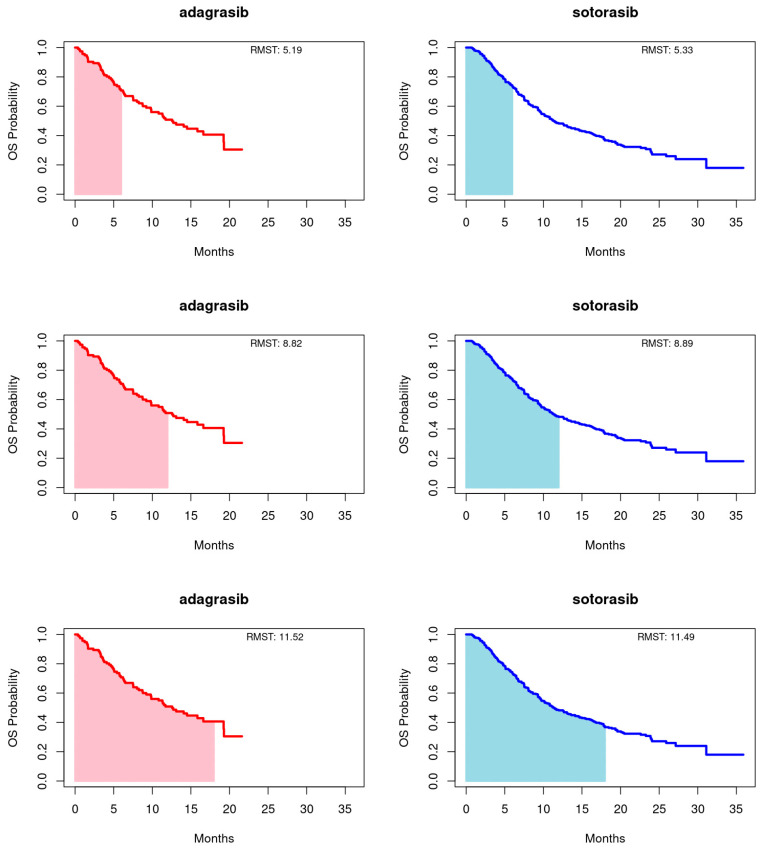
Restricted mean survival time (RMST) of overall survival (OS) for adagrasib (**left panel**) and sotorasib (**right panel**) was evaluated at 6 (**upper panel**), 12 (**middle panel**), and 18 (**bottom panel**) months.

Employing the RMST method, as proposed by Huang [22], we calculated the area under the Kaplan-Meier curves up to a predetermined timepoint, with a focus on the longer duration of the entire event-free distribution or the shorter follow-up curve, capped at 18 months for this study. Truncation times at 6 and 12 months were also utilized. This approach provides a comprehensive summary of the survival function, capturing the overall treatment effect across various timepoints. For PFS, adagrasib’s RMST consistently exceeded that of sotorasib at the 6-, 12-, and 18-month endpoints. Conversely, for OS, sotorasib slightly outperformed adagrasib at the 6- and 12-month marks, but adagrasib marginally surpassed sotorasib by the 18-month endpoint. It is important to note that the RMST differences between the treatments did not reach statistical significance at any timepoint for either PFS or OS, as illustrated in Figure 2 and Figure 3 and summarized in Table 3.

### 3.5. Clinical Relevance of Adverse Events

A notable difference between adagrasib and sotorasib is the prevalence of gastrointestinal side effects. In the KRYSTAL-1 trial, diarrhea (70.7%) and nausea (69.8%) were common with adagrasib. In contrast, CodeBreak100 and CodeBreak200 reported significantly lower rates for sotorasib, with diarrhea at 30% and 34% and nausea at just 14% in CodeBreak200. Patients with pre-existing gastrointestinal issues may benefit more from sotorasib, although most side effects were manageable and rarely led to treatment discontinuation. The tolerability profiles of adagrasib and sotorasib are crucial in clinical decision-making, especially when efficacy differences are minimal. Adagrasib’s higher gastrointestinal adverse events may deter its use in susceptible patients, while sotorasib’s more favorable safety profile—especially concerning Grade 3 or higher events—could make it more suitable for a wider patient range, particularly those with liver or gastrointestinal issues (Supplementary Table S1). Fatigue was another frequent adverse event. In KRYSTAL-1, 59.5% of adagrasib patients reported fatigue, while only 7% did in CodeBreak200 for sotorasib. This suggests that sotorasib may be better tolerated regarding energy levels, which is important for maintaining the quality of life. Clinicians should consider patient well-being when choosing between therapies. However, both drugs showed potential hepatotoxicity, indicated by elevated liver enzymes. In KRYSTAL-1, 28.4% of adagrasib patients experienced increased ALT levels, with 5.2% at Grade ≥ 3. Sotorasib showed lower elevations in CodeBreak100 (18%) and CodeBreak200 (10%), with 8% at Grade ≥ 3. The lower severe elevation rates with sotorasib may make it preferable for patients with liver concerns, as hepatotoxic effects were generally reversible.

Grade 3 or Higher Adverse Events: Adagrasib had a higher rate of Grade 3 or higher adverse events (81.9%) compared to sotorasib (20% in both CodeBreak100 and CodeBreak200). This resulted in more frequent dose reductions and interruptions in the adagrasib group (82.8% in KRYSTAL-1), while only 22% required such adjustments in CodeBreak100.

Serious Adverse Events: Both agents exhibited serious adverse events (SAEs), with adagrasib showing more gastrointestinal SAEs, while sotorasib was more associated with hepatotoxicity. Importantly, sotorasib had fewer severe gastrointestinal side effects, making it a better option for patients sensitive to these issues.

## 4. Discussion

The emergence of targeted therapies, particularly those addressing the KRAS G12C mutation, marks a significant advancement in the treatment landscape for NSCLC. This study aimed to juxtapose the efficacy of two FDA-approved therapies, adagrasib and sotorasib, by scrutinizing data from three seminal clinical trials: KRYSTAL-1, CodeBreak100, and CodeBreak200. Our analysis, grounded in reconstructed individual patient data, reveals nuanced differences in the performance of adagrasib and sotorasib over time. In terms of PFS, adagrasib consistently exhibited a higher RMST than sotorasib at the 6-, 12-, and 18-month benchmarks. This trend suggests a potentially more prolonged disease control with adagrasib in the initial treatment phases. However, the OS data present a more intricate scenario. Initially, sotorasib demonstrated a slight advantage, but adagrasib caught up and marginally exceeded sotorasib by the 18-month threshold.

The absence of statistical significance in the RMST disparities between the treatments for both PFS and OS indicates that while observable trends exist, they may not be sufficiently robust to singularly guide clinical decision-making. Nevertheless, these trends offer valuable insights for future research and clinical considerations. The Kaplan–Meier curves, particularly for PFS, corroborate that patients on adagrasib tend to experience a prolonged period before disease progression compared to those on sotorasib. This observation, coupled with the RMST analysis, highlights adagrasib’s potential in providing a more sustained response in PFS.

The transition from sotorasib’s initial OS superiority to adagrasib’s marginal lead at the 18-month mark prompts questions about the long-term impacts of these drugs and the possibility of differential benefits among patient subgroups. Further analyses, potentially focusing on specific patient characteristics or genetic markers, could elucidate these aspects. The consistent exclusion criteria across the trials, especially the exclusion of patients with prior KRAS G12C inhibitor treatment, lend credibility to our comparisons. However, this also implies that our findings may not extend to all NSCLC patients, particularly those with previous exposure to KRAS G12C inhibitors.

In trials assessing adagrasib and sotorasib, no significant differences in treatment efficacy or outcomes were found between male and female patients. The studies had a balanced gender distribution, with females representing 36.3% to 56.0% of participants. While female patients were somewhat under-represented in Dy 2023 [19], the diverse populations across all trials allowed for meaningful subgroup analyses. In the CodeBreaK100 trial (Dy 2023) [19], no statistically significant differences in efficacy outcomes, including overall survival or progression-free survival, were observed between genders. Similarly, the KRYSTAL-1 trial (Jänne 2022) [18] reported no notable differences in response rates between male and female patients. De Langen 2023 also found no specific gender-related efficacy trends for sotorasib compared to docetaxel. In conclusion, gender was considered in the demographic analyses, but current evidence indicates that both adagrasib and sotorasib provide similar clinical benefits for male and female patients. This suggests that these therapies are broadly effective for treating KRAS G12C-mutated NSCLC across genders. However, further studies with larger populations may shed light on any potential gender-related differences in treatment response.

As for subgroup analysis and trial heterogeneity, pooling data from the KRYS-TAL-1, CodeBreak100, and CodeBreak200 trials introduced heterogeneity due to variations in study designs, patient populations, and treatment regimens. As for subgroup analysis and trial heterogeneity, summary data from the KRYS-TAL-1, CodeBreak100, and CodeBreak200 trials introduced heterogeneity due to differences in study design, patient populations, and treatment regimens. Although both adagrasib and sotorasib have shown efficacy, potential differences in subgroup responses must be considered. The CodeBreak200 trial (De Langen 2023) [20] provided the most detailed subgroup analysis, examining factors such as age, ECOG performance status, smoking history, and PD-L1 expression in patients treated with sotorasib versus docetaxel. In terms of age, patients younger than 65 years responded slightly better to sotorasib than older patients, suggesting that age may influence treatment efficacy. The results were better although sotorasib was still effective in both groups of patients, suggesting its broader applicability, possibly due to differences in tumor biology. Finally, in terms of PD-L1 expression, patients with low or no PD-L1 expression (TPS < 1%) derived more benefit from sotorasib compared with docetaxel, whereas patients with high TPS (TPS ≥ 1%) showed a smaller differential benefit, indicating potential synergy with immune checkpoint inhibitors.

Despite the insights from De Langen 2023, the lack of detailed subgroup analyses in KRYSTAL-1 (Jänne 2022) [18] and CodeBreak100 (Dy 2023) [19] limits the ability to conduct a comprehensive pooled analysis. Both trials provide overall efficacy data but do not break down results by age, ECOG status, or PD-L1 expression. In conclusion, while the subgroup analysis suggests that factors like age, ECOG PS, smoking status, and PD-L1 expression can significantly impact the efficacy of KRAS G12C inhibitors, the absence of similar data from other trials makes it challenging to generalize these findings across all KRAS G12C treatments.

This study has several limitations. The reliance on reconstructed patient data, though a validated method, does not equate to the robustness of raw patient-level data. Moreover, pooling data from CodeBreak100 and CodeBreak200 for sotorasib might introduce a degree of heterogeneity, despite the trials’ similar designs and criteria. Additionally, the indirect comparison of drug effects due to varying patient cohorts warrants caution. Intriguingly, the CodeBreak100 trial indicated that patients with PD-L1 immunohistochemistry expression levels below 1% experienced more prolonged PFS benefits, a trend not mirrored in the CodeBreak200 trial. This discrepancy in PD-L1’s predictive value across trials necessitates further investigation to clarify its role in forecasting responses to KRAS G12C inhibitors.

The lack of consistent subgroup data across the three trials limits our ability to assess heterogeneity. Future studies with detailed subgroup analyses would enhance our understanding of the factors influencing the efficacy of KRAS G12C inhibitors, enabling more personalized treatment based on patient characteristics like age, performance status, and PD-L1 expression. Given the absence of significant differences in HRs and RMST for PFS and OS between adagrasib and sotorasib, clinical decision-making may increasingly focus on adverse reactions and overall drug costs. Shared decision-making between physicians and patients will be essential in this context.

## 5. Conclusions

Both adagrasib and sotorasib emerge as promising options for NSCLC patients harboring the KRAS G12C mutation. Our study underscores subtle yet noteworthy differences in their efficacy, with adagrasib potentially offering more sustained disease control in PFS. However, the OS benefits between the two drugs appear to converge over time. As the field of targeted NSCLC therapies continues to evolve, research endeavors like ours are vital in steering clinicians towards evidence-based treatment choices, ensuring optimal patient care. Future research should focus on the long-term effects of these drugs and the identification of patient subgroups that may derive greater benefit from one therapy over the other. However, many ongoing studies on KRAS G12C inhibitors will provide valuable insights into the future direction of targeted therapy for KRAS G12C-mutant NSCLC (Supplementary Table S2).

## Figures and Tables

**Table 1:** Comparative trial overview.

Study	Janne 2022	Dy 2023	De Langen 2023
Trial name	KRYSTAL-1	CodeBreak100	CodeBreak200
NCT number	NCT03785249	NCT03600883	NCT04303780
Trial phase	Phase II	Phase I/II	Phase III
Published arm(s)	One arm adagrasib 600 mg orally twice daily	One arm sotorasib 960 mg orally once daily	Two arms sotorasib 960 mg orally once daily docetaxel 75 mg/m^2^ intravenously once every 3 weeks
Inclusion criteria	1. Patients must be 18 years or older. 2. Must have a histologically confirmed diagnosis of unresectable or metastatic NSCLC with the KRASG12C mutation. 1. Must have previously received treatment with at least one platinum-containing chemotherapy regimen and checkpoint inhibitor line of therapy. 2. Must have measurable tumor lesions according to RECIST, version 1.1. 3. Must have an ECOG performance status score of 0 or 1.	1. Patients must be 18 years or older. 2. Must have pathologically documented, locally advanced, or metastatic NSCLC with the KRAS p.G12C mutation. 1. Disease progression after the receipt of anti-PD–1 or anti-PD-L1 immunotherapy, platinum-based chemotherapy, or both. 2. Must have an ECOG performance status score of 0 to 1. 3. Must have measurable disease according to RECIST, version 1.1.	1. Patients must be aged at least 18 years. 2. Must have histologically or cytologically documented, locally advanced, and unresectable or metastatic NSCLC, with the KRASG12C mutation. 1. Must have had tumor progression after receiving at least one previous systemic line of therapy for advanced disease. 2. Must have an ECOG performance status of 0–1. 3. Must have measurable disease according to RECIST, version 1.1. 4. Patients with treated, stable brain metastases were eligible.
Exclusion criteria	1. Active CNS metastases (eligible if adequately treated and neurologically stable). 2. Carcinomatous meningitis. 3. The receipt of systemic therapy or radiation therapy within 2 weeks before the first dose of adagrasib. 4. Previous treatment with a KRASG12C inhibitor.	1. Active untreated brain metastases. 2. The receipt of more than three previous lines of therapy. 3. The receipt of systemic anticancer therapy within 28 days before the initiation of sotorasib therapy. 4. The receipt of therapeutic or palliative radiation therapy within 2 weeks before the initiation of sotorasib therapy. 5. Previous treatment with a direct KRASG12C inhibitor.	1. New or progressing untreated brain lesions or symptomatic brain lesions. 2. A previously identified oncogenic driver mutation other than KRASG12C for which an approved therapy is available. 3. Previous treatment with docetaxel (neoadjuvant or adjuvant docetaxel allowed if no progression within 6 months after therapy termination). 4. Previous treatment with a direct KRASG12C inhibitor. 5. Systemic anticancer therapy within 28 days of study day 1. 6. Therapeutic or palliative radiation therapy within 2 weeks of treatment initiation.

**Table 2:** Comparative analysis of demographic and clinical characteristics in comparative trials.

Study	Janne 2022	Dy 2023	DeLangen 2023 (a)
Sample size—no.	116	174	171
Age, years—median (range)	64 (25 to 89)	65 (37 to 86)	64 (32 to 88)
Female sex—no. (%)	65 (56.0)	91 (52.3)	62 (36.3)
Race—no. (%)			
White	97 (83.6)	141 (81.0)	142 (83.0)
Asian	5 (4.3)	25 (14.4)	21 (12.3)
Black	9 (7.8)	4 (2.3)	2 (1.2)
Others	5 (4.3)	4 (2.3)	6 (3.5)
ECOG PS—no. (%)			
0	18 (15.5)	49 (28.2)	59 (34.5)
1	97 (83.6)	125 (71.8)	112 (65.5)
Data missing	1 (0.9)	0 (0.0)	0 (0.0)
Previous lines of therapy—no. (%)			
1	50 (43.1)	73 (41.9) (b)	77 (45.0)
2	40 (34.5)	58 (33.3)	65 (38.0)
≥2	26 (22.4)	43 (24.7)	29 (17.0)
Disease stage—no. (%)			
Locally advanced	13 (11.2)	6 (3.4)	9 (5.3)
Metastatic	103 (88.8)	168 (96.6)	162 (94.7)
Metastasis burden—no. (%)			
Bone	46 (39.7)	81 (46.6)	na
CNS	24 (20.7)	40 (23.0)	58 (33.9)
Liver	19 (16.4)	38 (21.8)	30 (17.5)
PD-L1 TPS/protein expression—no. (%)			
<1%	47 (40.5)	46 (26.4)	57 (33.3)
≥1% to <50%	27 (23.3)	42 (24.1)	46 (26.9)
≥50%	12 (10.3)	44 (25.3)	60 (35.1)
Data not available	30 (25.9)	42 (24.1)	8 (4.7)

Abbreviations: ECOG PS, Eastern Cooperative Oncology Group performance status; PD-L1 TPS, programmed death-ligand 1 tumor proportion score. nc, not calculable; na, not available. (a) Only the sotorasib arm. (b) Two patients with no prior line of therapy in phase I were included.

**Table 3:** MRST comparison of adagrasib and sotorasib. (a) PFS (b) OS.

**(a) PFS**			
**PFS**	**MRST**	**Lower**	**Upper**
6-month end			
adagrasib	4.677	4.331	5.022
sotorasib	4.514	4.317	4.711
adagrasib—sotorasib	0.163	−0.235	0.561
adagrasib/sotorasib	1.036	0.951	1.129
12-month end			
adagrasib	7.104	6.273	7.934
sotorasib	6.785	6.322	7.248
adagrasib—sotorasib	0.319	−0.632	1.270
adagrasib/sotorasib	1.047	0.914	1.199
18-month end			
adagrasib	8.556	7.257	9.854
sotorasib	8.077	7.397	8.757
adagrasib—sotorasib	0.479	−0.987	1.945
adagrasib/sotorasib	1.059	0.891	1.260
**(b) OS**			
**OS**	**MRST**	**Lower**	**Upper**
6-month end			
adagrasib	5.190	4.901	5.478
sotorasib	5.332	5.19	5.474
adagrasib—sotorasib	−0.143	−0.464	0.178
adagrasib/sotorasib	0.973	0.915	1.035
12-month end			
adagrasib	8.818	8.073	9.563
sotorasib	8.895	8.497	9.293
adagrasib—sotorasib	−0.077	−0.922	0.768
adagrasib/sotorasib	0.991	0.901	1.091
18-month end			
adagrasib	11.519	10.298	12.741
sotorasib	11.491	10.822	12.159
adagrasib—sotorasib	0.029	−1.364	1.421
adagrasib/sotorasib	1.002	0.888	1.131

## Data Availability

The data presented in this study are available in this article and Supplementary Materials.

## Supplementary Materials

The following supporting information can be downloaded at https://www.mdpi.com/article/10.3390/cancers16213676/s1, Table S1: The detailed comparison of the treatment-related adverse events (TRAEs) from these trials; Table S2: Current Landscape of KRAS G12C Inhibitors in Clinical Trials.
