# Supplementary material for: Comparative Efficacy of Adagrasib and Sotorasib in KRAS G12C-Mutant NSCLC: Insights from Pivotal Trials
Source: Cancers (Basel). 2024 Oct 30;16(21):3676. doi: 10.3390/cancers16213676 (PMC11545475; doi:10.3390/cancers16213676)
Supplement: Supplementary file 1 [file cancers-16-03676-s001.zip › cancers-3232153-supplementary.pdf]

Supplementary Files

Title: Comparative Efficacy of Adagrasib and Sotorasib in KRAS G12C-Mutant NSCLC: Insights from Pivotal Trials

Table S1. The detailed comparison of the treatment-related adverse events (TRAEs) from these trials.

Adverse Event	KRYSTAL-1 (Adagrasib) – Jänne 2022	CodeBreak100 (Sotorasib) – Dy 2023	deBreak200 (Sotorasib) – De Langen 2
Any adverse event (any grade)	100.00%	70%	Not reported
Diarrhea	70.70%	30%	34%
Nausea	69.80%	Not reported	14%
Vomiting	56.90%	Not reported	5%
Fatigue	59.50%	Not reported	7%
Anemia	36.20%	Not reported	3%
Elevated liver enzymes (ALT)	28.4% (Grade ≥3: 5.2%)	18%	10% (Grade ≥3: 8%)
Elevated liver enzymes (AST)	26.7% (Grade ≥3: 5.2%)	18%	10% (Grade ≥3: 5%)
Grade 3 or Higher AEs	81.90%	20%	20% (Sotorasib) / 47% (Docetaxel)
Serious Adverse Events (SAEs)	Diarrhea, nausea, fatigue	Hepatotoxicity	Hepatotoxicity, diarrhea

Table S2. Current Landscape of KRAS G12C Inhibitors in Clinical Trials.

Inhibitor	Clinical Trial Identifier	Phase	Indication	Efficacy (ORR / DCR)	Key Treatment-Related Adverse Events (TRAEs)	Reference
Divarasib (GDC-6036)	NCT04449874	Phase I	Advanced KRAS G12C-mutant NSCLC	ORR: 53.4% / DCR: Not reported	Rash, diarrhea, nausea, vomiting, dry skin, and paronychia	Sacher 2023
JDQ443	NCT04699188 (KontRASt-01)	Phase Ib/II	KRAS G12C-mutant solid tumors	ORR: 41.7% / DCR: Not reported	Fatigue, edema, diarrhea, nausea, vomiting, peripheral neuropathy	Lorthiois 2022
JAB-21822	NCT05009329 / NCT05288205	Phase I/II	KRAS G12C-mutant NSCLC	ORR: 70% (400 & 800 mg QD) / DCR: 100%	Anemia, bilirubin increase, proteinuria	Shi 2024
IBI-351 (GFH925)	NCT05005234	Phase I/II	KRAS G12C-mutant NSCLC	ORR: 61.2% / DCR: 92.5%	Anemia, decreased white blood cell count, ALT increases, pruritus	Yuan 2023
LY3537982	NCT04956640	Phase I	KRAS G12C-mutant NSCLC and solid tumors	ORR: 60% / DCR: 80%	Diarrhea, constipation, fatigue, peripheral edema, nausea, neutropenia	Hollebecque 2024
BI-1823911	NCT04973163	Phase I	KRAS G12C-mutant advanced or metastatic solid tumors	Early data: 3 confirmed partial responses (PRs)	Grade 3 diarrhea, gastrointestinal stoma complications, nausea, vomiting, 2 AEs led to discontinuation	Heymach 2023
HBI-2438	NCT05485974	Phase I	KRAS G12C-mutant	ORR: Early efficacy	No SAEs or DLTs in	Shojaei 2023

			NSCLC with brain metastasis	signals in brain metastasis; preclinical data show major tumor regression	early cohorts; well tolerated; combination therapy shows synergy with SHP2 inhibitors	
D-1553 (Garsorasib)	NCT05492045 / NCT04585035	Phase I/II	KRAS G12C-mutant NSCLC and solid tumors	ORR: 40.5% / DCR: 91.9%	Elevated AST/ALT, diarrhea, hypertension, hypokalemia, bilirubin increase, hypothyroidism	Li 2023, Li 2024
RMC-6291	NCT05462717	Phase I	KRAS G12C-mutant solid tumors	Preclinical ORR: 72% / Preclinical DCR: 92%	Dose escalation study ongoing; targeting the ON state of KRAS G12C mutations	Nokin 2024

References:

Yuan Y, Deng Y, Jin Y, Pan Y, Wang G, Wang Z, Zhang Z, Meng X, Hu Y, Zhao M, et al. Efficacy and safety of IBI351 (GFH925) monotherapy in metastatic colorectal cancer harboring KRAS G12C mutation: Preliminary results from a pooled analysis of two phase I studies. JCO. 2023;41(16_suppl):3586-3586.

Li Z, Dang X, Huang D, Jin S, Li W, Shi J, Wang X, Zhang Y, Song Z, Zhang J, et al. Garsorasib in patients with KRASG12C-mutated non-small-cell lung cancer in China: an open-label, multicentre, single-arm, phase 2 trial. The Lancet Respiratory Medicine. 2024;12(8):589-598.

Li Z, Song Z, Zhao Y, Wang P, Jiang L, Gong Y, Zhou J, Jian H, Dong X, Zhuang W, et al. D-1553 (Garsorasib), a Potent and Selective Inhibitor of KRASG12C in Patients With NSCLC: Phase 1 Study Results. Journal of Thoracic Oncology. 2023;18(7):940-951.

Nokin MJ, Mira A, Patrucco E, Ricciuti B, Cousin S, Soubeyran I, San José S, Peirone S, Caizzi L, Vietti Michelina S, et al. RAS-ON inhibition overcomes clinical resistance to KRAS G12C-OFF covalent blockade. Nat Commun. 2024;15(1):7554.

Shojaei F, Ricono JM, Fang C, Ning J, Lee G, Gillings M. Abstract A097: HBI-2438, HUYABIO selective KRASG12C inhibitor with BBB penetration, inhibited tumor growth in a metastatic brain model as single agent and also displayed synergy in combination with HBI-2376 (HUYABIO SHP2 inhibitor) in a CRC PDX model. Molecular Cancer Therapeutics. 2023;22(12_Supplement):A097-A097.

Heymach J, Kotecki N, Prenen H, Alonso G, Lindsay CR, Barve M, Thamer C, Eigenbrod-Giese S, Marotti MA, Van Lancker G. 665P First-in-human, phase Ia/b, dose-escalation/expansion study of KRAS G12C inhibitor BI 1823911, as monotherapy and combined with anticancer therapies, in patients (pts) with advanced or metastatic solid tumours harbouring a KRAS G12C mutation. Annals of Oncology. 2023;34:S468.

Hollebecque A, Kuboki Y, Murciano-Goroff YR, Yaeger R, Cassier AC, Heist RS, Fujiwara Y, Deming DA, Ammakkanavar N, Patnaik A, et al. Efficacy and safety of LY3537982, a potent and highly selective KRAS G12C inhibitor in KRAS G12C-mutant GI cancers: Results from a phase 1 study. JCO. 2024;42(3_suppl):94-94.

Lorthiois E, Gerspacher M, Beyer KS, Vaupel A, Leblanc C, Stringer R, Weiss A, Wilcken R, Guthy DA, Lingel A, et al. JDQ443, a Structurally Novel, Pyrazole-Based, Covalent Inhibitor of KRAS G12C for the Treatment of Solid Tumors. J Med Chem. 2022;65(24):16173-16203.

Sacher A, LoRusso P, Patel MR, Miller WH Jr, Garralda E, Forster MD, Santoro A, Falcon A, Kim TW, Paz-Ares L, et al. Single-Agent Divarasib (GDC-6036) in Solid Tumors with a KRAS G12C Mutation. N Engl J Med. 2023;389(8):710-721.

Shi Y, Fang J, Xing L, Y Yao, J Zhang, Liu L, Wang Y, Hu C, Xiong J, Liu Z, et al. A pivotal phase 2 single-arm study of glecirasib (JAB-21822) in patients with NSCLC harboring KRAS G12C mutation. JCO. 2024;42(36_suppl):468214-468214.
